# Data-driven classification of ordinary chondrites and asteroidal metal potential evaluation

**DOI:** 10.1038/s41598-026-35624-0

**Published:** 2026-01-20

**Authors:** Tian-Yu Liu, Si-Jia Wei, Ke-Li Shi, Tian-Qi Qiu, Jun-Zhe Teng, Zheng-Jie Qiu

**Affiliations:** 1 State Key Laboratory of Lithospheric and Environmental Coevolution, Institute of Geology and Geophysics, Chinese Academy of Sciences, Beijing, 100029 China; 2 College of Earth and Planetary Sciences, University of Chinese Academy of Sciences, Beijing, 100049 China; 3 Key Laboratory of Earth and Planetary Physics, Institute of Geology and Geophysics, Chinese Academy of Sciences, Beijing, 100029 China; 4 Aerospace Information Research Institute, Chinese Academy of Sciences, Beijing, 100094 China; 5 Maynooth International Engineering College, Fuzhou University, Fuzhou, 350116 China; 6 School of Ecology and Environment, Tibet University, Lhasa, 850000 China

**Keywords:** Ordinary chondrites classification, Machine learning, Bulk composition, Principal component analysis, Asteroidal metal potential evaluation, Planetary science, Solid Earth sciences

## Abstract

Ordinary chondrites (OCs) comprise ~ 87% of meteorites and are key to decoding early Solar System processes, yet H–L–LL classification is often ambiguous, especially between L and LL. We compile ~ 1100 published bulk analyses and train Support Vector Machine (SVM) and Random Forest (RF) classifiers on 13 geochemical features (including Si-normalized ratios and trace elements). Both models achieve an overall accuracy of 0.90, with precision of 0.96–0.97 (H), 0.86–0.89 (L), and 0.71–0.80 (LL), outperforming traditional single-proxy discrimination. Feature importance is dominated by Fe/Si and Ni/Si, consistent with metal–silicate fractionation trends. Principal component analysis (PCA) shows strong covariance among Fe–Ni–Co and inverse correlations with Si–Mg–Ca, separating metal-rich H from silicate-rich L–LL. To link taxonomy with resource assessment, we propose a Metal Potential Index (MPI) based on (Fe/Si + Ni/Si + Co/Si) after max-normalization; group means decrease from H (1.23) to L (0.87) to LL (0.75). Complementary statistical tests indicate that Fe–Ni (with Co admixtures) show limited dependence on petrographic type within each chemical group, supporting a near-uniform distribution of FeNi (Co) grains at the parent-body scale. Together, ML, PCA, and MPI provide a reproducible, data-driven framework for OC classification and for ranking asteroidal metal potential, identifying H-type parent bodies as the highest-priority metal targets.

**Supplementary Information:**

The online version contains supplementary material available at 10.1038/s41598-026-35624-0.

## Introduction

 Ordinary chondrites (OCs) comprise ~ 87% of meteorite finds and falls and thus anchor much of our understanding of early Solar System processes^1,2^. Multiple, independent lines of evidence link OCs to inner–main-belt S-type asteroids^3–11^: (i) spectral linkage, asteroid reflectance spectra (after space-weathering correction) closely match laboratory spectra of OCs; (ii) dynamical delivery, inner-belt resonances efficiently deliver S-type fragments to near-Earth space, matching OC fall statistics; and (iii) sample-return ground truth, returned particles (e.g., Itokawa dust) exhibit OC-like mineralogy and chemistry, confirming compositional affinity. This three-strand linkage underpins comparative planetology and, critically, provides a practical scaffold for asteroid in-situ resource utilization (ISRU) target prioritization and cost–benefit analysis.

Chemical group classification of OCs underpins reconstructions of parent-body differentiation, collisions, and dynamical evolution and, crucially, guides evaluations of asteroidal resources for ISRU. OCs are typically classified into three chemical groups: high-iron (H), low-iron (L), and low-iron-low-metal (LL)^1^, based on the amount of total iron, of iron metal and iron oxide in the silicates. In practice, boundaries among H, L, and LL groups can overlap in common geochemical spaces (e.g., Fe–Mg–Si)^1,12–14^, and many samples—particularly small fragments, weathered finds, or legacy specimens—lack the mineralogical (Fa–Fs) or oxygen-isotope (Δ^17^O) data required by traditional schemes. From a resource perspective, a longstanding question is whether the abundance of Fe–Ni–Co alloy varies systematically with petrographic type or is effectively uniform at the parent-body scale—an outcome that would allow S-type asteroids to be treated as geologically coherent, metal-bearing deposits with limited internal heterogeneity aside from localized impact overprints^15–23^. Because sample-return missions are definitive but costly and rare^24^, there is a clear need for inexpensive, scalable methods that extract diagnostic information directly from bulk chemistry to (i) assign OC group membership and (ii) provide first-order ISRU metrics prior to mission selection. Here we apply supervised machine learning (ML) to bulk compositions to deliver reproducible OC group classifications and a quantitative basis for estimating metal resource potential.

As large, heterogeneous datasets have become commonplace, ML has emerged as a practical integrator for geoscience and planetary data^25,26^. Traditional OC classification—built on a handful of geochemical indicators or mineralogical/oxygen-isotope criteria—often cannot be applied to small, weathered, or legacy samples and does not scale well to multidimensional patterns. Non-destructive approaches such as magnetic susceptibility measurements provide an efficient and widely used alternative for classifying ordinary chondrites, especially for small or curated samples^27^. However, magnetic susceptibility can also be influenced by weathering, oxidation of Fe–Ni metal, shock-induced microstructural changes, and heterogeneous metal distribution, potentially complicating interpretations for altered or fragmentary materials.

In contrast, ML learns nonlinear decision rules from many features simultaneously, yielding reproducible, uncertainty-aware classifications^25,28–31^. Our contribution is not ML itself, but its first systematic, bulk-chemistry-only application to OC classification at scale: we assemble a harmonized database (~ 1100 analyses), engineer Si-normalized ratios, address class imbalance, and train cross-validated SVM/RF models. To connect model behavior to process, we pair permutation importance with PCA to reveal metal–silicate fractionation signals, and we introduce a Metal Potential Index (MPI) that maps OC group identity onto ISRU-relevant metal potential. Together, these steps close a key gap—providing robust group identification and first-order resource screening when Fa–Fs and Δ^17^O data are unavailable. Therefore, our approach is not intended to replace mineralogical or magnetic methods, but rather to server as a scalable and data-driven framework that extends OC classification to broader datasets and supports comparative analyses across diverse sample populations.

Here we address both needs with a unified, data-driven framework. From ~ 1100 published OC bulk compositions, we train supervised classifiers (SVM, RF) on Si-normalized major/trace features to yield robust, reproducible group assignments with interpretable feature rankings. We then use principal component analysis (PCA) to reveal the dominant covariance structure—metallic Fe–Ni–Co versus silicate-forming Si–Mg–Ca—and introduce a Metal Potential Index (MPI), defined as the max-normalized sum of Fe/Si, Ni/Si, and Co/Si (MPI = Σ[(X/Si)/max(X/Si)]). In tandem, ML delivers high-accuracy classification, PCA clarifies geochemical controls consistent with near-uniform FeNi (Co) distributions at asteroid scale, and MPI provides an operational first-pass screen for ISRU targeting across H–L–LL parent bodies.

## Results

### Machine learning classification

**Fig. 1:**
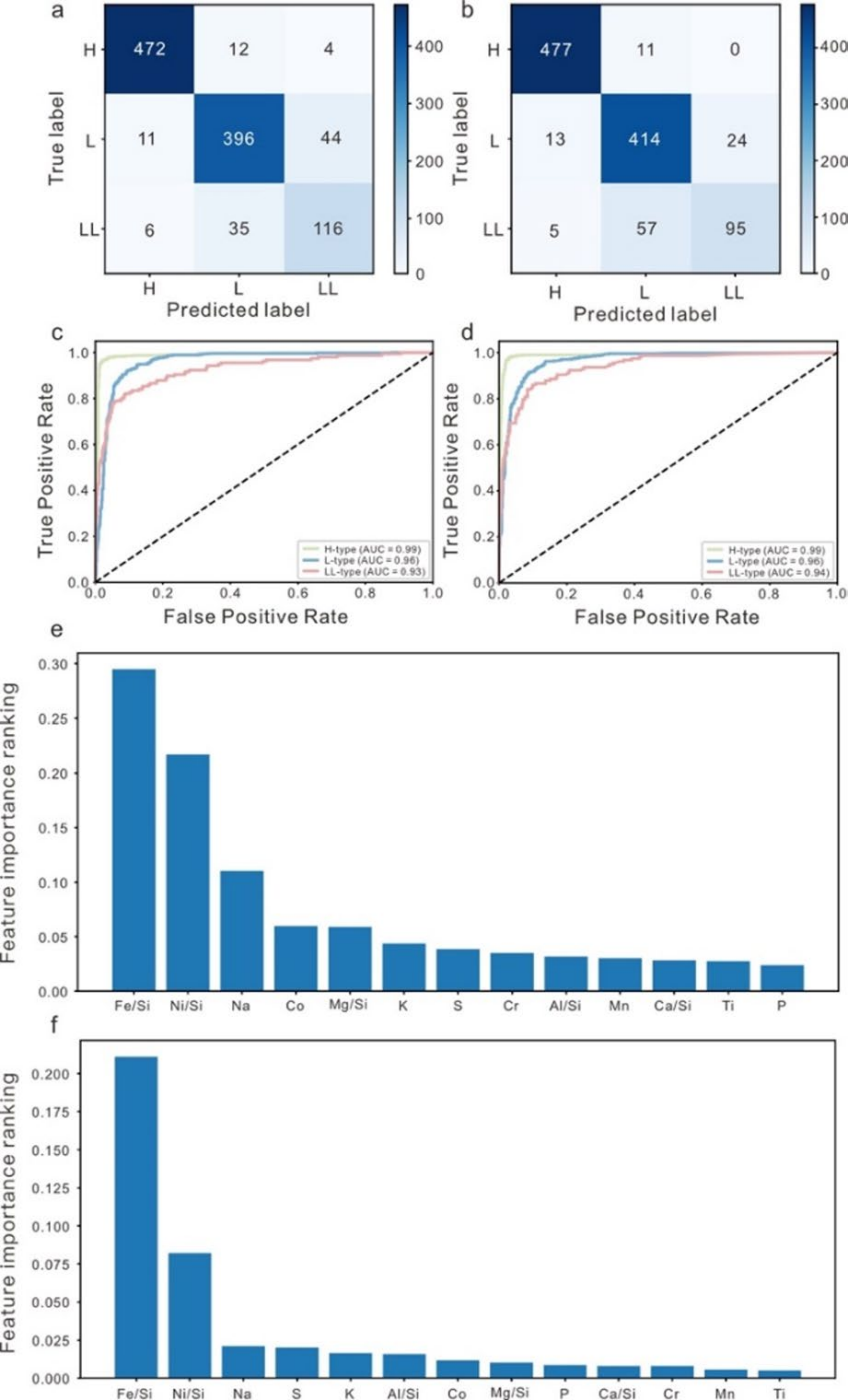
Model performance metrics and feature importance derived from machine learning classifiers applied to ordinary chondrite classification. (**a**, **b**) Confusion matrices illustrating the prediction accuracy of the support vector machine (SVM) and random forest (RF) classifiers, respectively, on the test dataset. (**c**, **d**) Receiver operating characteristic (ROC) curves with corresponding area under the curve (AUC) values for the SVM and RF models. (**e**) Relative feature importance for the RF classifier estimated using permutation importance scores. (**f**) Relative feature importance for the SVM classifier estimated using permutation importance scores. The rankings highlight the relative contributions of individual elemental features to the classification performance across chondrite groups.

Average performance metrics for each classifier from the tenfold cross-validation process with individual metrics for each fold are summarized in Supplementary Data Tables S1 and S2. The results of tenfold cross-validation show that two classifiers have higher stability. Two ML classifiers are applied to the test set, and their performance metrics are reported in Fig. 1a,b; Table 1. The SVM classifier performs well, as indicated by overall accuracy (0.90), precision (0.71–0.97), recall (0.74–0.97), and F1-score (0.72–0.97) scores. For the RF classifiers, all the metrics scores are also high (mostly > 0.80) with accuracy being 0.90. All these metric scores indicate that two supervised ML classifiers are robust in predicting OC group. Both classifiers also show high AUC values (0.96 for SVM and 0.96 for RF) (Fig. 1c,d). All these metric scores indicate that two supervised ML classifiers are robust in predicting OC groups.

In each method, the prediction performance for the OCs group from H and L chondrites is always better than that from LL chondrites. For example, for the H and L chondrites, the precision scores are almost all above 0.85 (average 0.92); in contrast, they range from 0.71 to 0.80 (average 0.75) for LL chondrites. This indicates that LL chondrites are slightly more difficult to be correctly distinguished compared to H and L chondrites, which is consistent with previous observations. To evaluate the contribution of each elemental feature to the classification of OCs, we utilized a trained RF classification model to rank feature importance. As illustrated in Fig. 1e, Fe/Si and Ni/Si exhibit the highest importance scores, followed by Na, Co, and Mg/Si, with other elements contributing progressively less (i.e., K, S, Cr, Al/Si, Mn, Ca/Si, Ti, and P). While SVM are less interpretable in terms of direct feature importance, we employed permutation importance^32^ to estimate the relative contribution of each variable. The results for the SVM model, shown in Fig. 1f, similarly identify Fe/Si and Ni/Si as the dominant features, with other elements playing minor roles. The consistency in feature importance rankings across both RF and SVM models highlights the robustness of Fe/Si and Ni/Si as key discriminators among OC groups. This convergence further validates the potential of elemental composition in accurately classifying OCs and supports the reliability of the machine learning-based approach.

**Table 1:** Summary of the performance metric scores for different ML classifiers.

Method	Type	Accuracy	Precision	Recall	F1-score
SVM	H	0.90	0.97	0.97	0.97
	L		0.89	0.88	0.89
	LL		0.71	0.74	0.72
RF	H	0.90	0.96	0.98	0.97
	L		0.86	0.92	0.89
	LL		0.80	0.61	0.69

### Principal component analysis and metal potential index

**Fig. 2:**
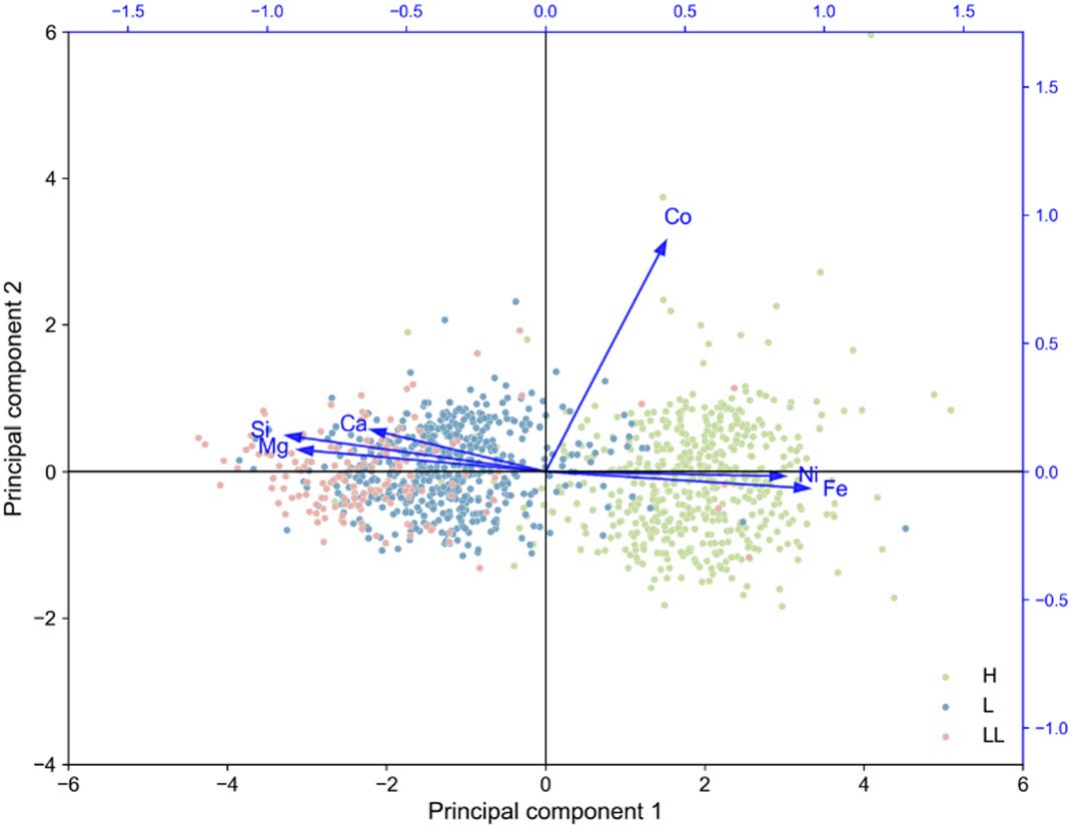
PCA graphic representation of principal components (PC2 vs. PC1).

Two principal components (PC1 and PC2) were identified, explaining 78.77% of the total variance (Table 2). Their eigenvalues are 3.867 and 0.863, corresponding to 64.40% and 14.38% of the variance, respectively. As shown in Table 3, Fe (0.946) and Ni (0.859) have strong positive loadings on PC1, while Mg (− 0.888) and Si (− 0.931) load negatively, clearly separating metal-rich and silicate-rich compositions. Co (0.897) is the dominant variable on PC2, and Ca shows moderate correlations with both components. The PCA plot (Fig. 2) illustrates that H chondrites cluster on the right, associated with high Fe, Ni, and Co, whereas L and LL chondrites lie on the left, enriched in Si, Mg, and Ca. PC2 represents minor variations mainly linked to Co and Ca. Overall, PC1 effectively distinguishes the metal-rich H group from the silicate-rich L–LL groups, confirming compositional differences among ordinary chondrites. This trend is consistent with the machine learning classification, where H chondrites show better discrimination performance than L and LL types.

**Fig. 3:**
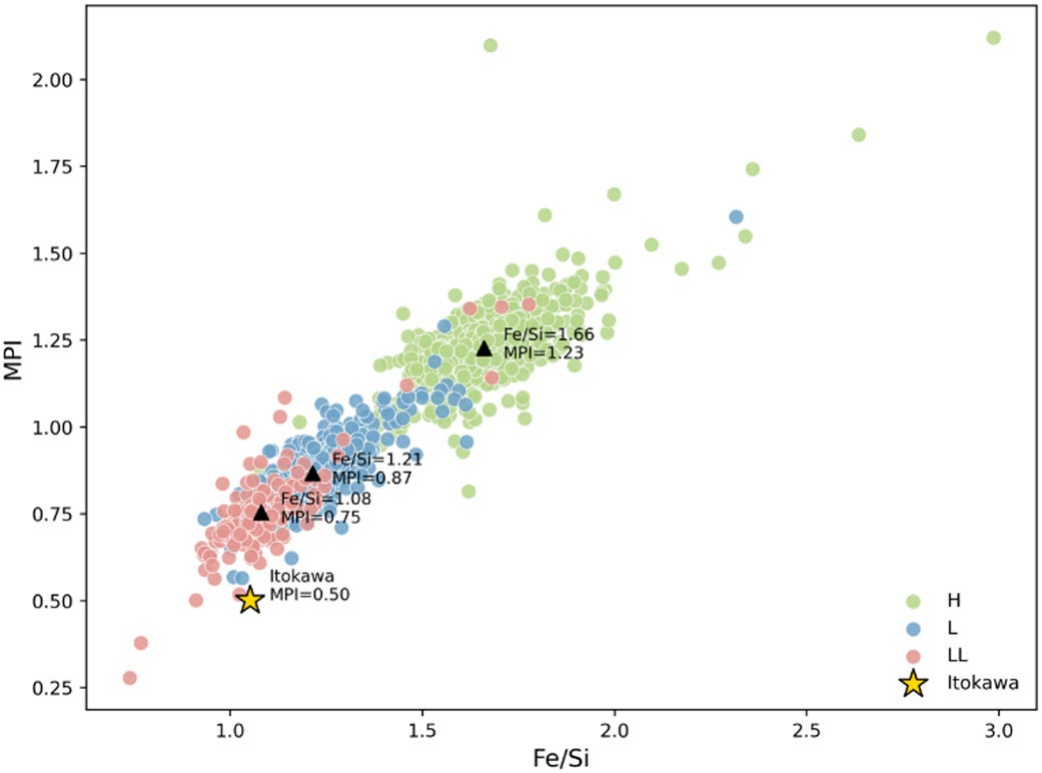
Relationship between the Metal Potential Index (MPI) and the Fe/Si ratio. The bulk composition of asteroid 25,143 Itokawa is taken from Ebihara et al. (2011) and Nakamura et al. (2011)^7,33^.

The calculated metal potential index (MPI) values show clear differences among the three ordinary chondrite groups. The H chondrites have the highest average MPI (1.23), followed by the L group (0.87) and the LL group (0.75), indicating a systematic decrease in metallic potential from H to LL types. In the MPI–Fe/Si plot (Fig. 3)^7,33^, all groups display a strong positive correlation, with H chondrites clustering in the upper right field and LL chondrites in the lower left. The Itokawa asteroid plots close to the LL field, with an MPI value of 0.50, suggesting a relatively low metallic resource potential consistent with its LL-like composition.

**Table 2:** The eigenvalues of the PCA analysis.

Principal components	Eigenvalue	Percentage of variance (exceeding 10%)	Cumulative variance (%)
1	3.867	64.40	64.40
2	0.863	14.38	78.77

**Table 3:** Factor loadings of both extracted PCs (PC1 and PC2).

Element	Factor loading PC1	Factor loading PC2
Mg	− 0.888	0.086
Si	− 0.931	0.141
Ca	− 0.626	0.164
Fe	0.964	− 0.065
Co	0.432	0.897
Ni	0.859	− 0.018

## Discussion

Supervised ML offers a powerful framework for handling complex, multidimensional geochemical datasets; however, its performance is highly contingent upon the volume and quality of input data^34,35^. While previous studies have utilized SVM and logistic regression (LR) to classify ordinary chondrite groups, these methods have faced limitations stemming from low spectral resolution, class imbalance, and sensitivity to data preprocessing procedures^36,37^. In this study, we employed ML algorithms to classify ordinary chondrites using bulk elemental compositions. The models achieved an overall accuracy of 0.90 for both SVM and RF under ten-fold cross-validation, with precision of 0.96–0.97 for H, 0.86–0.89 for L, and 0.71–0.80 for LL. In practice, predictions of LL should be flagged for targeted isotopic or mineralogical confirmation. These results indicate that multivariate elemental data carry strong discriminative signals and provide a complementary in-laboratory tool when Fa–Fs and Δ^17^O data are unavailable.

Although the classification models for ordinary chondrite chemical groups developed in this study demonstrate high validation accuracies, several limitations must be acknowledged regarding their broader applicability. First, despite extensive efforts to compile all currently available bulk chemical datasets on OCs from published literature and public repositories, the volume of high-quality analyses—particularly for LL chondrites—remains limited. This scarcity may hinder the models’ ability to generalize across underrepresented classes. Second, the present classification framework is restricted to the three principal chemical groups (H, L, and LL), without accommodating intermediate or transitional groups (e.g., H/L or L/LL) that are frequently reported in the literature and often defy clear categorization using traditional geochemical methods^38,39^. These studies reveal a diversity of solar system materials higher than previously assumed, mirroring the differences in physicochemical conditions in the solar nebula and structures of meteorite parent bodies^40^. The challenges faced in classifying LL-type chondrites, as evidenced by their relatively lower precision, and recall values in ML models, may partly stem from their greater susceptibility to thermal alteration and shock metamorphism, which can homogenize chemical signatures and complicate group distinctions. This further underscores the importance of considering asteroidal overprinting when interpreting geochemical data, particularly for borderline or mixed-group samples (e.g., L/LL). Third, despite the overall robustness of model performance metrics, classification errors persist, most notably between L and LL group, due to their overlapping geochemical characteristics. This underscores the need for cautious interpretation when applying the models to single samples and highlights the importance of incorporating multiple analyses or representative datasets to enhance classification reliability. Fourth, OCs may exhibit significant internal compositional heterogeneity resulting from complex thermal histories or post-accretionary alteration processes—variations that may not be fully captured by the current models. Finally, terrestrial weathering can alter bulk elemental compositions by oxidizing metallic Fe and sulfides to Fe²⁺/Fe³⁺ oxides and hydroxides (e.g., goethite, akaganéite)^40,41^. Such processes blur the compositional distinctions among H, L, and LL chondrites, introducing additional scatter in bulk Fe–Mg–Si relationships and thereby increasing classification uncertainty for some weathered samples. These considerations should be considered in future work, particularly when applying machine learning-based classifiers to newly recovered or poorly characterized meteorite specimens.

Beyond these methodological considerations, it is equally important to place our classification findings within the broader cosmochemical context of ordinary chondrite formation and evolution. Meteorites provide us with both a great diversity of extraterrestrial materials and a unique insight into early Solar System processes^42^. The majority of the chemical and mineralogical characteristics of OCs were established through processes that took place within the solar nebula over a brief interval during the early stages of solar system formation^43^. Many studies have shown patterns of chemical fractionations in chondrites relative to the solar composition to understand formative processes and reasons for the differences between chondrite classes^43–45^. The results of this study reveal that among the 13 geochemical features selected for machine learning classification, Fe/Si and Ni/Si are consistently the most significant variables in distinguishing H, L, and LL groups of OCs (Fig. 1e, f). These features not only dominate the classification model’s predictive power but also carry key information about the evolutionary processes that occurred in the solar nebula and on their parent bodies.

**Fig. 4:**
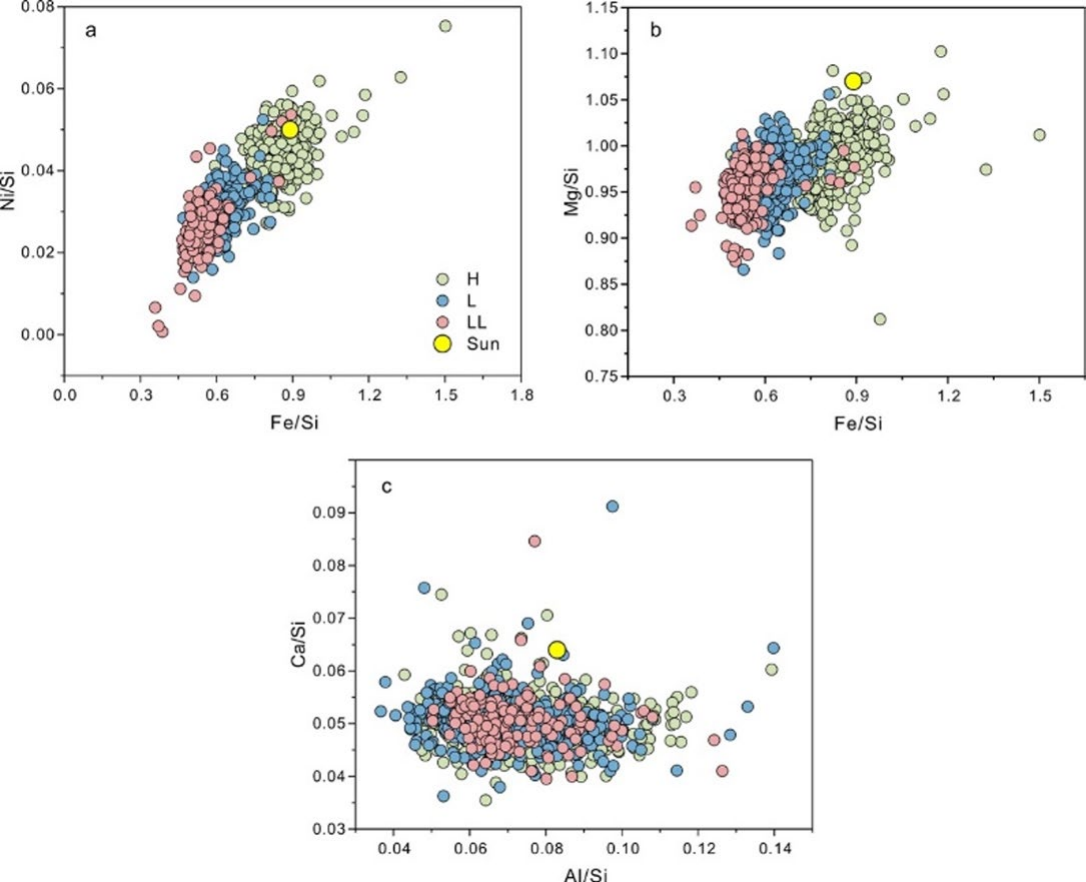
Abundances of the Fe, Ni, Mg, Ca and Al normalized to Si, in the three OC classes and the solar photosphere. Abundances in OCs in Fig. 4 is from Supplementary Data 2. Photospheric abundances are from^48^.

As illustrated in Fig. 4a, a pronounced depletion trend in Fe, closely correlated with Ni, is observed from the solar composition to H, L, and LL chondrites. A similar progressive decrease is also evident in Fe and Si contents. This pattern reflects the relative proportions of metallic and silicate phases and serves as a sensitive indicator of metal–silicate fractionation within the solar nebula^46^. The Fe-Ni depletion trajectory suggests that metallic components were progressively removed, leaving behind a residual dust assemblage increasingly enriched in silicates and depleted in metal^43^. The well-defined compositional gradient from H to LL chondrites in bulk Fe/Si ratios—where LL chondrites exhibit up to ~ 50% Fe depletion relative to solar composition—supports the hypothesis of systematic Fe-Ni metal loss during successive chondrule-forming shock events^45^. These events likely triggered partial melting and facilitated the physical segregation of immiscible metal droplets from silicate melts, a mechanism corroborated by petrographic evidence from CR chondrites^47^. In addition to metal-silicate segregation, oxidation also exerted a significant influence, with oxidation degrees negatively correlated with metal-silicate fractionation, leading to variations in Mg/Si ratios (Fig. 4b)^46,48,49^. LL chondrites, exhibiting the highest Δ¹⁷O values among OCs, became the most oxidized and isotopically heavy group due to extensive phyllosilicate formation from nebular water. In contrast, H chondrites remained the most reduced and isotopically light. Progressive metamorphism induced dehydration of phyllosilicates, releasing additional oxidizing agents, although direct evidence for partially dehydrated phyllosilicates in type-4 chondrites remains lacking^46^.

In contrast, refractory elements such as Ca, Al, and their respective ratios (Ca/Si, Al/Si), although included in the classification model, were ranked much lower in importance. Their near-uniform depletion (~ 20%) across all chondrite groups (Fig. 4c), as indicated in previous studies^50^, suggests that an early, high-temperature process—possibly driven by energetic shock waves—selectively removed Ca– and Al-rich components from the system. This process appears decoupled from the later, Fe/Ni-driven evolutionary trend. The machine learning models corroborate this interpretation, as Ca, Al, and related ratios were among the least predictive features, implying limited variability among OC groups in these elements.

The smooth compositional trajectory from solar to H, L, and LL groups—observed in the multi-dimensional elemental space—is not merely an artifact of classification, but rather reflects a physically plausible evolutionary sequence. The fractionation pattern supports the hypothesis that three discrete ordinary chondrite parent bodies formed sequentially along a metal-depletion trend, and that each accretion event was curtailed when the body was dynamically perturbed out of the nebular midplane. This interpretation explains the lack of continuous material mixing between groups and accounts for the distinct clustering of chemical compositions observed in both natural data and classification outputs.

The PCA results reveal a strong covariance among metallic elements (Fe, Ni, Co) and an inverse correlation with silicate-forming elements (Si, Mg, Ca), reflecting the effects of metal–silicate fractionation in ordinary chondrite (OC) parent bodies. The nearly uniform distribution of FeNi (Co) alloys inferred from the PCA is consistent with observations of present-day asteroids^16^. Regardless of whether the internal structure follows an onion-shell or rubble-pile model, individual fragments of these bodies show minimal variation in metallic content. Such compositional uniformity suggests that asteroids can be considered geologically coherent, metal-bearing deposits with homogeneously distributed FeNi(Co) grains, providing a foundation for assessing extraterrestrial ore potential^2,16^.

The Metal Potential Index (MPI) offers a quantitative metric for evaluating the metallic resource potential across OC groups. The calculated MPI values decrease systematically from H (1.23) to L (0.87) and LL (0.75) chondrites, indicating a progressive depletion in Fe–Ni–Co alloys and a corresponding enrichment in silicate phases. Samples with MPI values exceeding 1.23 represent metal-rich parent bodies with higher exploration potential. This finding aligns with resource distribution models of OC parent asteroids, in which Co- and Ni-bearing alloys are homogeneously dispersed throughout both regolith and interior materials^14,16^. Consequently, H chondrite parent bodies represent the most promising targets for in-situ metal extraction due to their elevated MPI and internally uniform metallic distribution.

In summary, our study demonstrates that machine learning applied to bulk major–trace element compositions provide a rapid and robust framework for distinguishing OC chemical groups. When combined with PCA and the MPI, this approach not only enhances classification accuracy but also reveals the geochemical and resource implications of metal–silicate fractionation. The PCA indicates that metallic phases are uniformly distributed across asteroidal fragments, while the MPI quantitatively ranks their metal potential, identifying H chondrite parent bodies (MPI > 1.23) as the most favorable exploration targets. Rather than replacing traditional mineralogical–isotopic criteria, this integrated framework harmonizes classification and resource evaluation, offering new insights into nebular differentiation, asteroidal evolution, and extraterrestrial resource utilization.

## Methods

### Data compilation and feature selection

We compiled more than 20,000 OC bulk element analyses from published studies (the data collected from the cited references are listed in the reference list of the Supplementary Data) and meteorite database (such as MetBase, https://www.lpi.usra.edu/meteor/metbull.php and Astromat Astromaterials DataSystem, https://astromat.org/), for which the group are known. For each OC analysis, 21 features, including Si, Ti, Al, Cr, Fe, Mn, Mg, Ca, Na, K, P, Ni, Co, S and 7 derived element ratios (Al/Si, Mg/Si, Ca/Si, Fe/Si, Ni/Si, Fe/Mg and Ni/Mg), were initially compiled. These ratios reflect metal-silicate fractionation, which have been suggested to be related to both nebular and asteroidal processes^46^. We did not compile features like siderophile-elements (Os, Ir, Au, As, Ga, Sb, Se and Zn), Co concentration in kamacite and Fa content in olivine, as well as O isotopic composition, which may also be useful in the identification of the group of OCs^1,12,13,51,52^, since these values are often not reported.

**Fig. 5:**
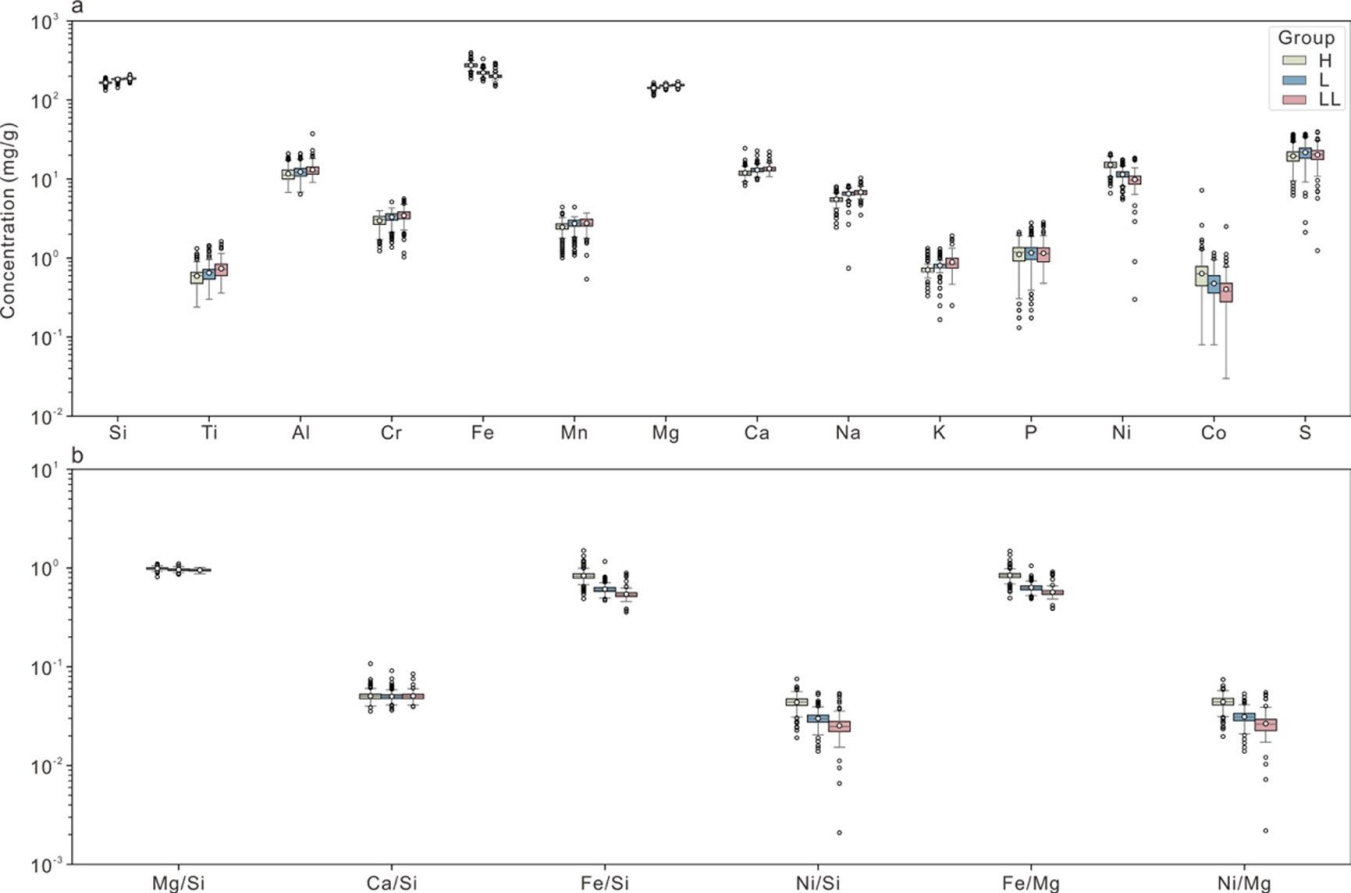
Box and whisker plots of 21 major and trace element concentrations and/or ratios for H, L, and LL group OC. The height of the colored bars represents the interquartile range. The horizontal black lines within the colored bars are the median and the open circles with black edges represent the mean value. “Whiskers” of each box illustrate the maximum values lying within 1.5 times the interquartile range beyond the edges of the bars. The colored crosses represent the outliers deviating by more than ± 1.5 σ. The data points outside the box are all higher than the average because of the use of log transformation.

The compiled 21 elements and/or ratios have many advantages in their application to ML modeling. First, they are routinely analyzed in many laboratories and are more commonly reported in literature studies (using the ICP-MS, ICP-ES, XRF and standard wet chemical analysis method). We assume that reliable and comparable data have been obtained since 1969 due to the development of reliable and modern analytical methods. Second, they have been shown to be useful in discriminating OC group, despite some claims to that there is still some difficulty in distinguishing L from LL^1,12–14^. Moreover, our statistical analysis work has indicated that although none of these selected elements and/or ratios can independently identify all three types of OCs, most can distinguish at least one OC group from the rest (Fig. 5). For example, most OCs from H group can be distinguished from the other two groups by higher Fe, Ni, Fe/Si, Ni/Si, Fe/Mg and lower Na, most OCs from L group can be distinguished by middle Fe, Fe/Si and Ni/Si, and most OCs from LL group can be distinguished by much higher Fe, Fe/Si and Ni/Si (Fig. 5). It can be also observed that many elements/ratios can readily distinguish the H group from the other two groups. However, the distinction between the L and LL group is relatively complex, as there is considerable overlap in the values of many elements between them. This observation is consistent with previous studies^14^.

**Fig. 6:**
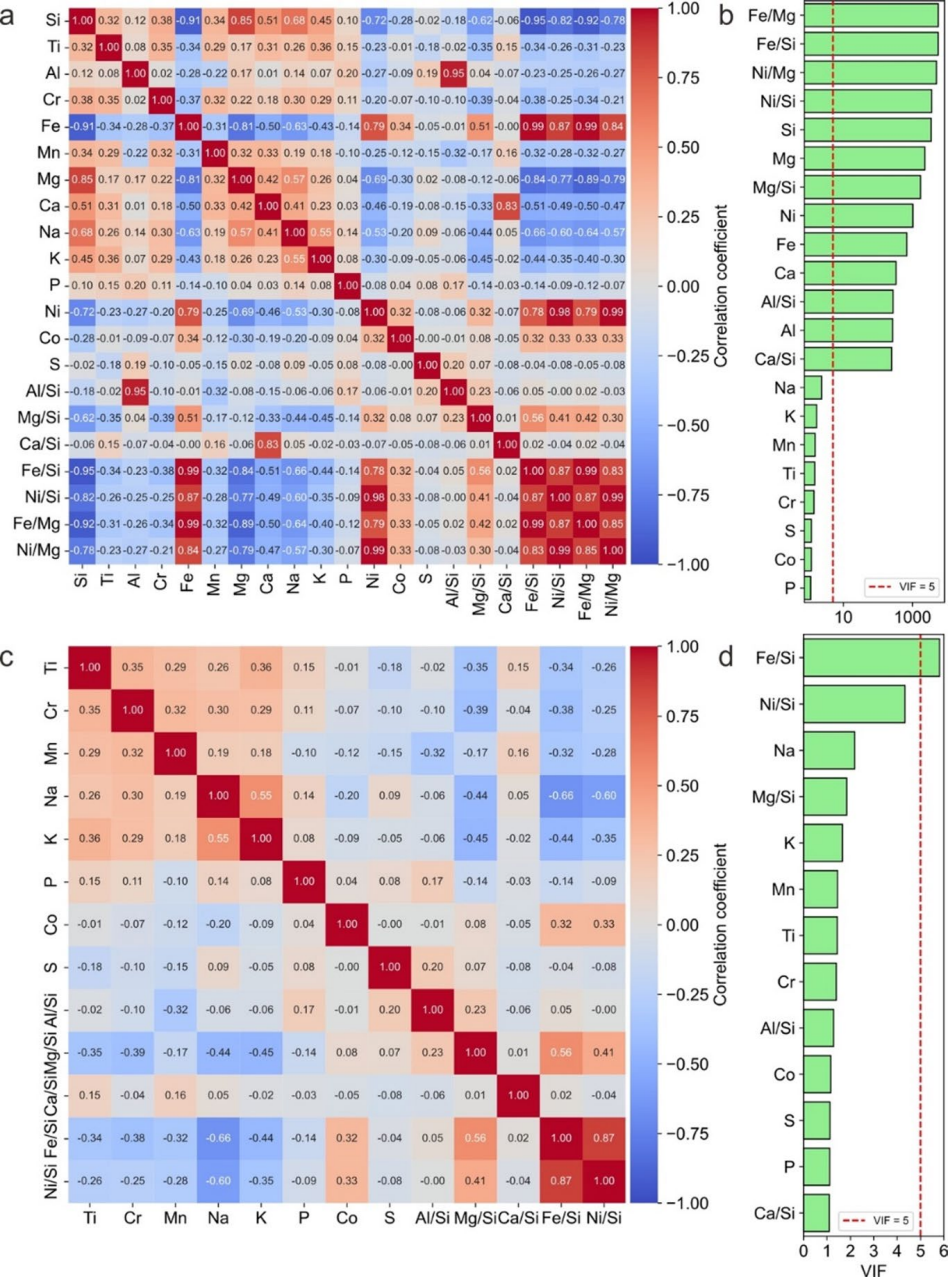
Parameters used to identify the multicollinearity of OCs features.

For each OC analysis, 21 geochemical features were initially compiled. However, not all of these features were employed during the ML training process. This decision was based on numerous studies indicating that including a larger number of features does not necessarily lead to improved model performance^52,53^. On the contrary, excessive features may introduce additional noise, thereby increasing the likelihood of misclassification or inaccurate predictions^54,55^. A common underlying cause of reduced performance is multicollinearity, wherein several features exhibit strong correlations not only with the dependent variable but also among themselves^56,57^. This issue can distort model outcomes and compromise the interpretability of individual feature effects, as changes in one feature inevitably affect correlated features^58^. Consequently, models affected by multicollinearity may yield unreliable or misleading conclusions. To enhance model performance and interpretability, careful feature selection is therefore essential. In this study, we applied both the correlation matrix method^59^ and the variance inflation factor (VIF)^56^ to evaluate multicollinearity among the 21 compiled features. The correlation matrix quantifies pairwise linear correlations among input features, with commonly used thresholds for multicollinearity ranging from 0.6 to 0.8^56^. Correlation coefficients exceeding these thresholds suggest a significant risk of multicollinearity. In parallel, the VIF is a widely used diagnostic tool for detecting multicollinearity in regression models^59,60^. It measures how much the variance of a regression coefficient is inflated due to collinearity among independent features^58^. A VIF < 5 indicates negligible multicollinearity, values between 5 and 10 suggest moderate multicollinearity, while VIF > 10 implies severe multicollinearity and unreliable coefficient estimation^56,60^. Figure 6 displays the heatmap of the correlation matrix and the VIF for the 21 features. Both confirm that element ratios (Al/Si, Mg/Si, Ca/Si, Fe/Si, Ni/Si, Fe/Mg and Ni/Mg) and elements (Si, Fe, Mg, Ni, Al, and Ca) are highly correlated and have a significant multicollinearity problem. According to the correlation matrix (Fig. 6a) and VIF values (Fig. 6b), a total of 13 features were ultimately selected for the ML modeling: Fe/Si, Ni/Si, Mg/Si, Al/Si, Ca/Si, Na, K, Mn, Ti, Cr, Co, S, and P. The recalculated correlation matrix and VIF values (Fig. 6c,d) confirm that no significant multicollinearity exists among these features, with the exception of Fe/Si and Ni/Si, which exhibit a certain degree of correlation. Previous principal component analyses (PCA) have identified Fe as the most representative element among Fe, Ni, and Co in OCs^14^. However, Ni/Si is considered to reflect metal-silicate fractionation and redox variations during the early condensation stages of the solar nebula^46,61^. Accordingly, Fe/Si and Ni/Si were retained in the final feature set due to their geochemical significance.

### Treatment of missing values and data filtering

According to the above descriptions, the ML modeling conducted in this study requires a minimum of 13 features, including 8 elemental concentrations and 5 elemental ratios. However, not all of these 13 features were available for every analysis due to missing values in the compiled database. This limitation is unavoidable, as the dataset was assembled from multiple sources employing varying analytical techniques, many of which did not report the complete suite of elements. Despite this, the proportion of missing data in the final dataset is minimal (< 0.02%). To address these gaps, mean imputation was applied, wherein missing values were replaced with the mean value of the corresponding feature across the entire dataset. Analyses with more than three missing values were excluded from further consideration. It is well established that geochemical datasets rarely conform to normal or log-normal distributions^62^ and statistical outliers may arise naturally due to the heterogeneous nature of geological processes. Therefore, in this study, we did not exclude statistical outliers, as doing so might introduce bias into the model. Instead, we focused on identifying and mitigating the influence of outliers that result from analytical or human error.

**Fig. 7:**
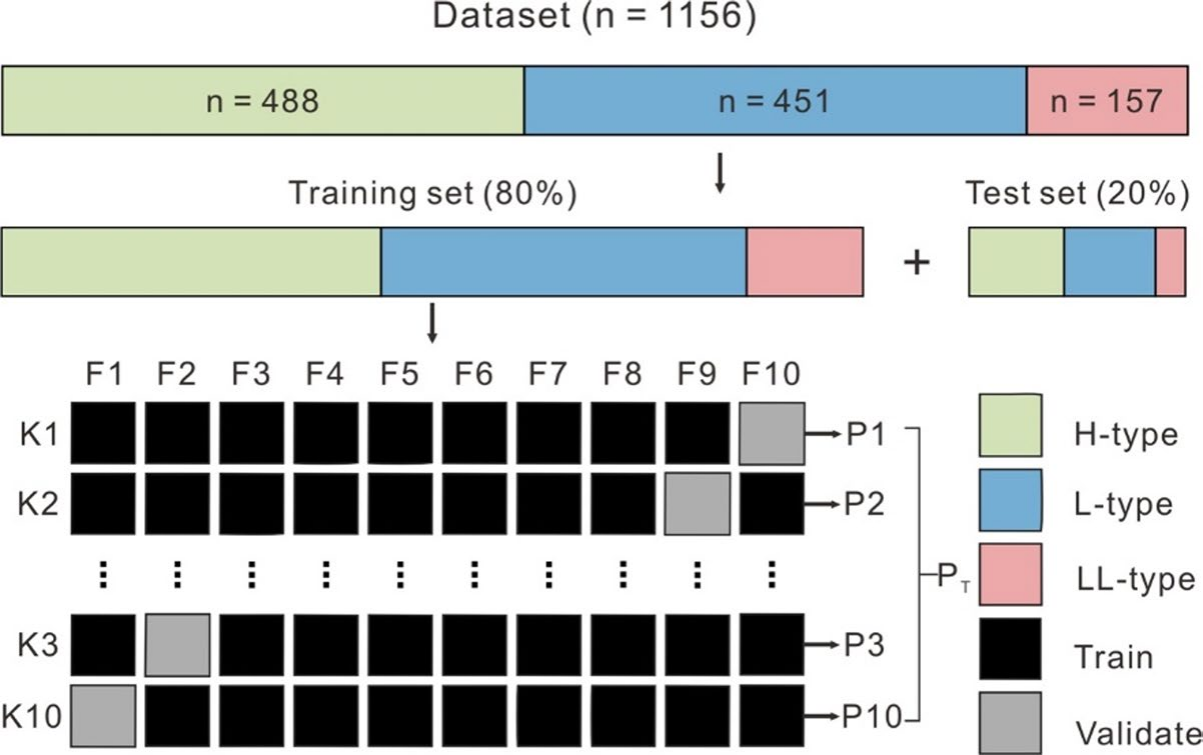
Data splitting scheme and schematic illustration of a tenfold cross-validation workflow (modified after^53,63^. The dataset is split into a training set and a test set. The training set is further divided tenfold. On this basis, ten times of cross-validation are performed, with one of them selected as a validation set for evaluation in each training. Performance metrics (P) are calculated for each fold and the mean metric (P_T_) is calculated as the overall performance. The proportion of each class remains the same throughout the training.

Subsequently, the datasets for H, L, and LL chondrites were randomly divided into training (80%) and test (20%) sets (Fig. 7)^63^, while maintaining the original distribution of high and low values for each feature across both subsets.

### Treatment of class imbalance

In this study, the compiled OCs analyses from different groups are imbalanced: the proportion of LL group (14%) is noticeably lower than that from H group (43%) and L group (43%). This is because LL chondrites have the least abundant of the OCs, and the data from experiments conducted on them are also more limited. Such a class imbalance is a common problem in ML. Such class imbalance is a common challenge in ML, and previous studies have shown that most classifiers tend to be biased toward the majority classes, resulting in poor classification performance for minority classes^64^. To address this issue, commonly used strategies include oversampling the minority class or undersampling the majority class to construct a more balanced dataset. In this study, we applied an undersampling approach, as our preliminary tests indicated it yielded better performance metrics than oversampling. Specifically, we employed the Tomek Link undersampling technique^65^. The advantage of this method lies in its focus on removing borderline and noisy instances rather than enforcing a strict balance between classes, thereby preserving the overall integrity of the dataset^65^. This helps reduce the risk of discarding valuable information—an issue often associated with conventional undersampling methods^66^.

After applying this technique, 488 samples from the H group, 451 from the L group, and 157 from the LL group were retained for further analysis (Fig. 7). All data sources are detailed in Supplementary Data 2. While the resulting dataset may not be perfectly balanced or exhaustive, it represents the most comprehensive compilation available to the authors at the time of manuscript preparation.

### Machine learning methods

ML algorithms have been widely applied to address classification problems in the geosciences^25^. Compared to traditional methods, ML offers advantages in identifying and mitigating bias while enhancing the reproducibility of scientific analyses^25^. Among the various ML techniques, SVM and RF are two of the most commonly used algorithms in geoscience applications^29,67^. In this study, we apply both SVM and RF to classify ordinary chondrites based on their bulk geochemistry. Using these two distinct algorithms enables a comparative evaluation of classification accuracy and model interpretability. SVM is well-suited for small to medium datasets with high-dimensional features but can be sensitive to parameter tuning. RF, an ensemble method, is more robust to overfitting and provides insights into feature importance. By combining both approaches, we aim to enhance classification reliability and assess their respective advantages and limitations. The core principles of SVM and RF are outlined below.

**Fig. 8:**
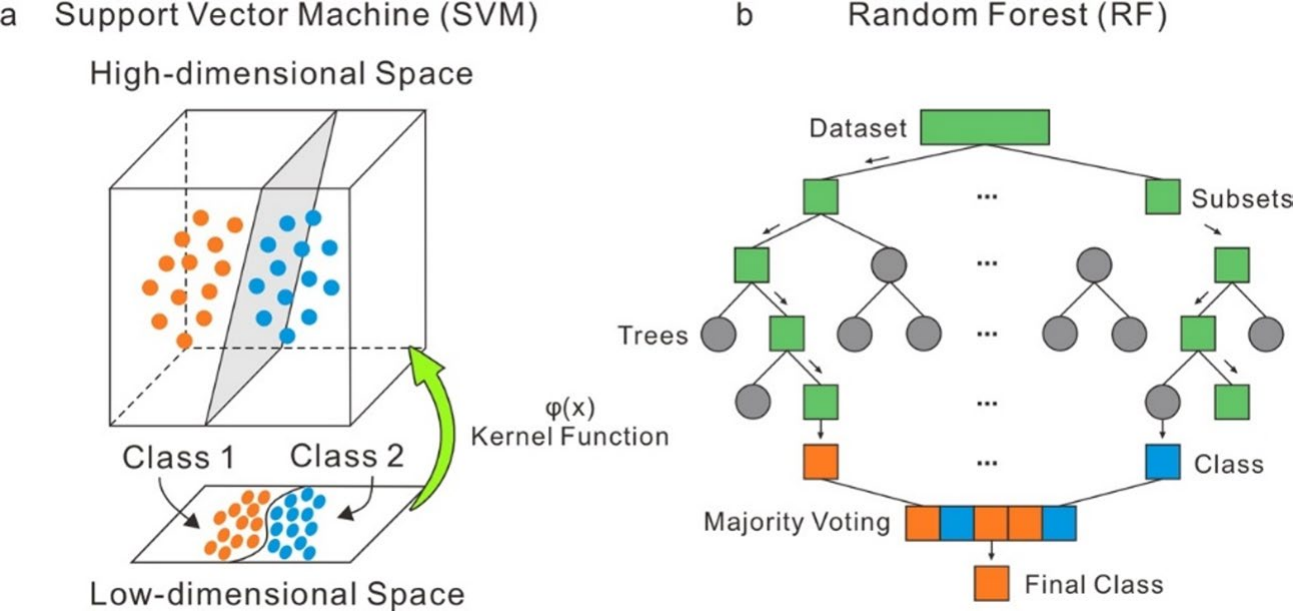
Schematic illustrations of the two supervised ML classifiers used in this study (modified after^53^).

SVM is a supervised learning algorithm grounded in statistical learning theory and the principle of structural risk minimization^68^. Its core objective is to construct an optimal hyperplane in the feature space that maximally separates different classes^69^. For datasets that are not linearly separable, SVM employs kernel functions to map input data from a low-dimensional space into a higher-dimensional space, in which linear separation becomes feasible (Fig. 8a). A more detailed explanation of the mathematical foundation of SVM is available in^70,71^.

RF is a robust ensemble learning algorithm introduced by^72^. Ensemble learning combines multiple weak classifiers to form a stronger, more accurate classifier. RF operates as an averaging algorithm that constructs a multitude of decision trees, each trained on a randomly selected subset of samples and features (Fig. 8b). The training subsets for each tree are generated using the bootstrap aggregating (bagging) method, which samples with replacement from the original dataset to maintain the same sample size^72^. Each decision tree casts a vote for classification, and the class receiving the majority of votes is selected as the final prediction. For a more comprehensive overview of the RF classifier, see^73^.

ML algorithms are parameterized, and their performance can be significantly influenced by the choice of hyperparameters. These hyperparameters are not directly learned from the data but must be specified prior to model training^67^. To optimize the performance of the two ML algorithms used in this study, a grid search approach combined with tenfold cross-validation was employed to identify the best set of hyperparameters^74^.

For SVM and RF, two and five hyperparameters, respectively, were subjected to optimization (Supplementary Data Figs. S2, S3). The optimal hyperparameter combinations determined by grid search were selected for final model implementation (Supplementary Data Table S3). The classifiers trained with these optimized parameters were subsequently evaluated on the test set to determine their final performance.

**Fig. 9:**
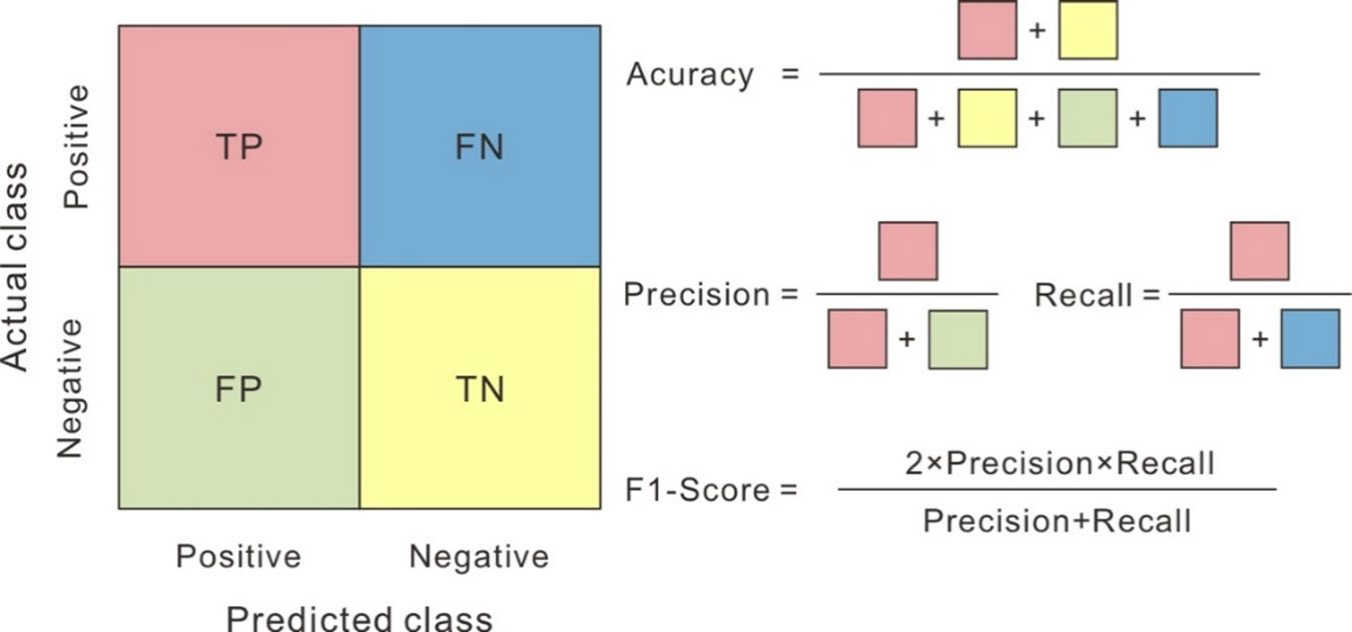
Schematic diagram of the calculation of the four metrics (accuracy, precision, recall, and F1-score) based on the confusion matrix (left). TP (true positive) is the number of positive samples predicted correctly. FP (false positive) is the number of positive samples predicted incorrectly. TN (true negative) is the number of negative samples predicted correctly. FN (false negative) is the number of negative samples predicted incorrectly. Accuracy, precision, recall, and F1-score range from 0 to 1; theoretically, if the samples from the test set are all predicted correctly, accuracy, precision, recall, and F1-score would be 1.

Model performance was assessed using four standard classification metrics: accuracy, precision, recall, and F1-score. These metrics are derived from the confusion matrix, which reports the number of true positives (TP), true negatives (TN), false positives (FP), and false negatives (FN) (Fig. 9). Accuracy is the proportion of correctly classified samples relative to the total number of samples. Precision measures the proportion of correctly predicted positive samples among all samples predicted as positive. Recall is the proportion of correctly predicted positive samples relative to all actual positive samples. F1-score is the weighted harmonic mean of precision and recall, providing a balanced measure of a model’s classification performance.

### Principal component analysis

To determine whether the distribution of Fe and Ni (with Co admixtures) is independent of petrographic types within particular groups of chondrites. We analyzed principal chemical components (Si, Mg, Ca, Fe, Ni, and Co) in OCs based on our database. The PCA toolkit enables a new representation of data from the input data set while minimizing information loss^75^. PCA provides a representation of variables, which allows finding variables that are characteristic of specific sample groups. It also allows better understanding of the nature of data and indicating the factors of their variability^76^.

### Metal potential index

The metal potential index (MPI) was used to evaluate the relative metallic resource potential of ordinary chondrites and their parent asteroids. MPI was calculated from bulk elemental ratios of Fe, Ni, and Co normalized to Si, representing the major metallic components relative to silicate material. To minimize compositional bias, each ratio was normalized to its maximum value within the dataset before summation, following:

$${\mathrm{MPI}}={\left( {{\mathrm{Fe}}/{\mathrm{Si}}} \right)_{\mathrm{n}}}+{\left( {{\mathrm{Ni}}/{\mathrm{Si}}} \right)_{\mathrm{n}}}+{\left( {{\mathrm{Co}}/{\mathrm{Si}}} \right)_{\mathrm{n}}}$$

where (X/Si)_n_ = (X/Si) / max(X/Si). Higher MPI values indicate higher concentrations of metallic elements relative to silicates, implying greater in-situ metal extraction potential.

## Supplementary Information

Below is the link to the electronic supplementary material.

Supplementary Material 1

Supplementary Material 2

## Data Availability

All data used in this study are derived from previously published sources and publicly accessible databases. Bulk chemical compositions of ordinary chondrites were compiled from literature^12,41,77^ and from established meteorite databases, including the Meteoritical Bulletin Database (https://www.lpi.usra.edu/meteor/metbull.php) and the Astromat Astromaterials DataSystem (https://astromat.org/). The compiled dataset is provided in the Supplementary Data 2. No new data were generated for this study.
