# Supplementary material for: Data-driven classification of ordinary chondrites and asteroidal metal potential evaluation
Source: Sci Rep. 2026 Jan 20;16:5826. doi: 10.1038/s41598-026-35624-0 (PMC12894755; doi:10.1038/s41598-026-35624-0)
Supplement: Supplementary file 1 — Supplementary Material 1 [file 41598_2026_35624_MOESM1_ESM.docx]

**Supplementary Data**

**Contents of this file**

Figures S1 to S3

Tables S1 to S3

References cited in the Supplementary Materials


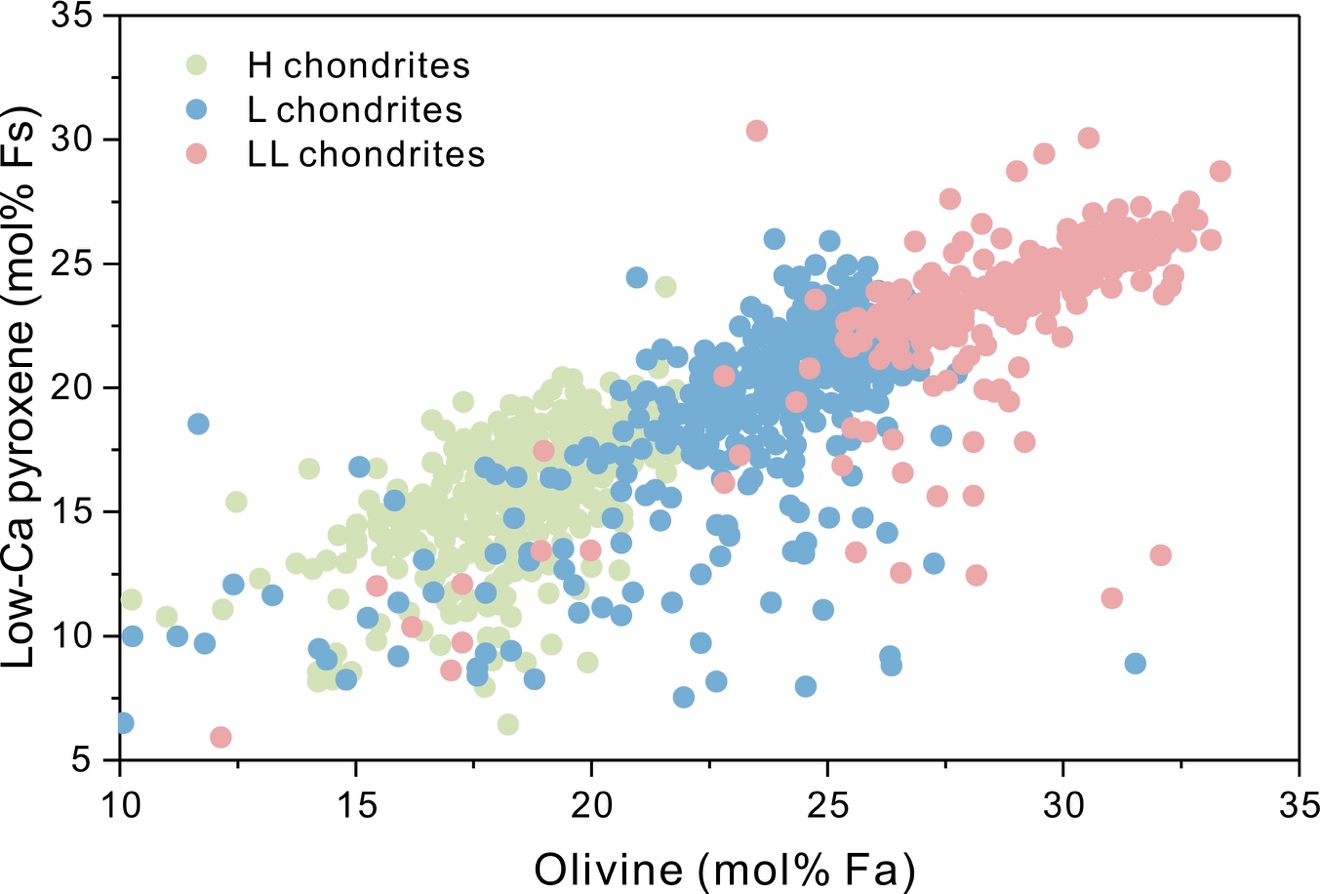


**Fig. S1.** Fa-Fs Classification Diagram for the ordinary chondrites. The pentagram represents the data for the TnA 001 Chondrite, while data for L, LL, and H groups are taken from Nakamura et al. (2011) and Demidova et al. (2022).


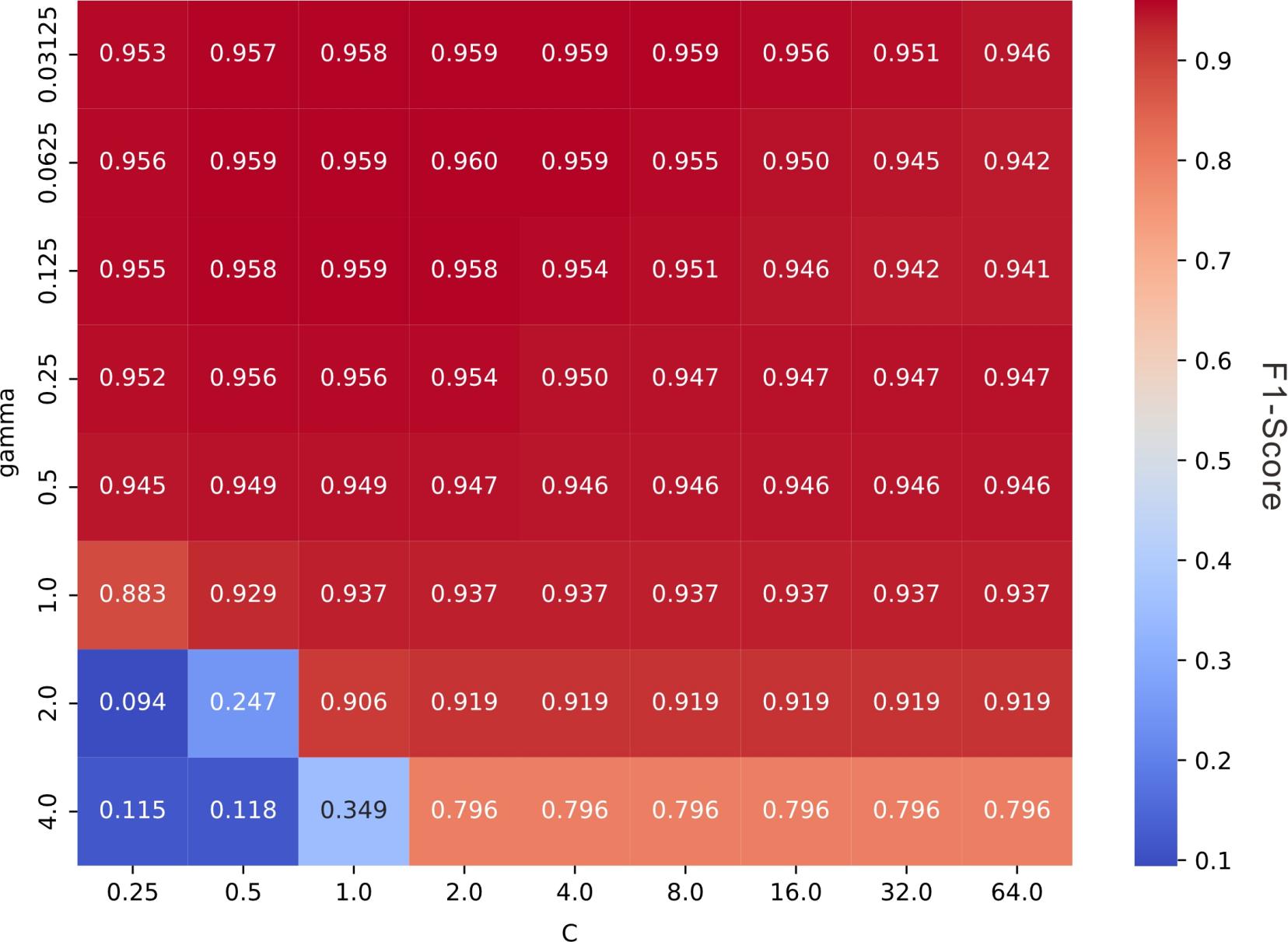


**Fig. S2.** Grid search to optimize C and gamma for SVM, with a tenfold cross validation technique used as a performance metric.


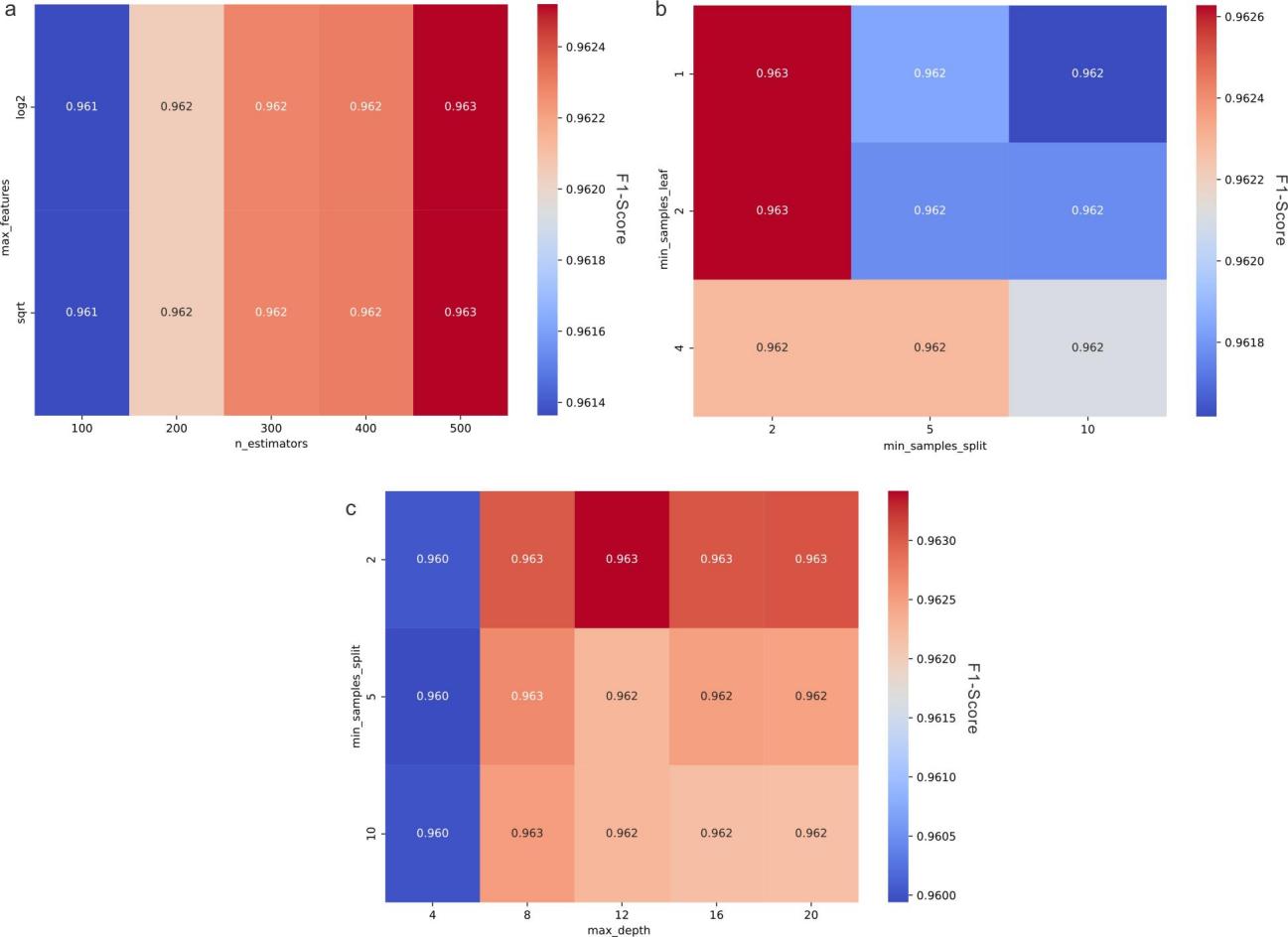


**Fig. S3.** Grid search to optimized parameters for RF, with a tenfold cross validation technique used as a performance metric. a. Parameter optimization for max_features and n_estimators. b. Parameter optimization for min_samples_leaf and min_samples_split. c. Parameter optimization for min_samples_split and max_depth .

**Table S1.** Performance metrics for SVM using 10-fold cross validation.

| Fold | Accuracy | Precision | Recall | F1-Score | AUC |
| --- | --- | --- | --- | --- | --- |
| 1 | 0.94 | 0.93 | 0.88 | 0.90 | 0.95 |
| 2 | 0.87 | 0.82 | 0.84 | 0.83 | 0.96 |
| 3 | 0.87 | 0.84 | 0.85 | 0.84 | 0.94 |
| 4 | 0.89 | 0.84 | 0.84 | 0.84 | 0.96 |
| 5 | 0.91 | 0.87 | 0.89 | 0.88 | 0.98 |
| 6 | 0.90 | 0.87 | 0.87 | 0.87 | 0.96 |
| 7 | 0.89 | 0.86 | 0.85 | 0.85 | 0.94 |
| 8 | 0.92 | 0.90 | 0.86 | 0.87 | 0.97 |
| 9 | 0.90 | 0.85 | 0.88 | 0.86 | 0.98 |
| 10 | 0.89 | 0.83 | 0.86 | 0.84 | 0.97 |
| Mean | 0.90 | 0.86 | 0.86 | 0.86 | 0.96 |
| Std | 0.02 | 0.03 | 0.02 | 0.02 | 0.02 |

**Table S2.** Performance metrics for RF using 10-fold cross validation.

| Fold | Accuracy | Precision | Recall | F1-Score | AUC |
| --- | --- | --- | --- | --- | --- |
| 1 | 0.92 | 0.91 | 0.84 | 0.86 | 0.97 |
| 2 | 0.91 | 0.88 | 0.86 | 0.87 | 0.97 |
| 3 | 0.85 | 0.81 | 0.78 | 0.79 | 0.93 |
| 4 | 0.92 | 0.92 | 0.85 | 0.88 | 0.97 |
| 5 | 0.92 | 0.90 | 0.88 | 0.89 | 0.99 |
| 6 | 0.90 | 0.88 | 0.87 | 0.87 | 0.96 |
| 7 | 0.89 | 0.89 | 0.82 | 0.85 | 0.94 |
| 8 | 0.87 | 0.83 | 0.78 | 0.79 | 0.97 |
| 9 | 0.93 | 0.90 | 0.85 | 0.87 | 0.98 |
| 10 | 0.90 | 0.85 | 0.82 | 0.83 | 0.97 |
| Mean | 0.90 | 0.88 | 0.84 | 0.85 | 0.97 |
| Std | 0.02 | 0.04 | 0.04 | 0.03 | 0.02 |

**Table S3** Optimal parameters for different ML methods.

| Model | Hyper-parameter | Value | Note |
| --- | --- | --- | --- |
| SVM | kernel | rbf |  |
|  | C | 0.03125 | A regularization parameter that permits some resilience in spatial overlaps between classes, as a part of the optimization problem to illustrate or define these hyperplanes |
|  | gamma | 1 | A regularization parameter that is related to maximizing model performance and minimizing model complexity. Large values of gamma imply that the model would be little regulated and more nonlinear |
|  | class_weight | balanced | A parameter for adjusting the weights associated with classes to solve the imbalanced data problem |
| RF | n_estimators | 500 | The number of decision trees |
|  | min_samples_split | 2 | The number of samples required for segmentation |
|  | max_depth | 12 | The maximum depth of the tree |
|  | min_samples_leaf | 2 | The minimum number of samples required at leaf nodes |
|  | max_features | sqrt | The maximum number of features that can be used by a single decision tree |
|  | class_weight | balanced | A parameter for adjust the weights associated with classes to solve the imbalanced data problem |

**REFERENCES**

Demidova, S. I., Whitehouse, M. J., Merle, R., Nemchin, A. A., Kenny, G. G., Brandstätter, F. et al. A micrometeorite from a stony asteroid identified in Luna 16 soil. Nat. Astron. 6, 560–567 (2022).

Kallemeyn, G. W., Rubin, A. E., Wang, D. & Wasson, J. T. Ordinary chondrites: Bulk compositions, classification, lithophile-element fractionations and composition–petrographic type relationships. Geochim. Cosmochim. Acta 53, 2747–2767 (1989).

Kimura, M., Imae, N., Yamaguchi, A., Haramura, H. & Kojima, H. Bulk chemical compositions of Antarctic meteorites in the NIPR collection. Polar Sci. 15, 24–28 (2018).

Haramura, H., Kushiro, I. & Yanai, K. Chemical compositions of Antarctic meteorites I. Mem. Natl. Inst. Polar Res. Spec. Issue 30, 109–121 (1983).

Meteoritical Bulletin Database. https://www.lpi.usra.edu/meteor/metbull.php (2025).

Astromat Astromaterials DataSystem. https://astromat.org/ (2025).
